# Comparative Genomics of the Zoonotic Pathogen *Ehrlichia chaffeensis* Reveals Candidate Type IV Effectors and Putative Host Cell Targets

**DOI:** 10.3389/fcimb.2016.00204

**Published:** 2017-01-25

**Authors:** Christophe Noroy, Damien F. Meyer

**Affiliations:** ^1^CIRAD, UMR ASTREGuadeloupe, France; ^2^INRA, UMR 1309 ASTREMontpellier, France; ^3^Université des AntillesGuadeloupe, France

**Keywords:** type IV effectors, *Ehrlichia chaffeensis*, comparative genomics, host-pathogen interactions, genome plasticity

## Abstract

During infection, some intracellular pathogenic bacteria use a dedicated multiprotein complex known as the type IV secretion system to deliver type IV effector (T4E) proteins inside the host cell. These T4Es allow the bacteria to evade host defenses and to subvert host cell processes to their own advantage. *Ehrlichia chaffeensis* is a tick-transmitted obligate intracellular pathogenic bacterium, which causes human monocytic ehrlichiosis. Using comparative whole genome analysis, we identified the relationship between eight available *E. chaffeensis* genomes isolated from humans and show that these genomes are highly conserved. We identified the candidate core type IV effectome of *E. chaffeensis* and some conserved intracellular adaptive strategies. We assigned the West Paces strain to genetic group II and predicted the repertoires of T4Es encoded by *E. chaffeensis* genomes, as well as some putative host cell targets. We demonstrated that predicted T4Es are preferentially distributed in gene sparse regions of the genome. In addition to the identification of the two known type IV effectors of *Anaplasmataceae*, we identified two novel candidates T4Es, ECHLIB_RS02720 and ECHLIB_RS04640, which are not present in all *E. chaffeensis* strains and could explain some variations in inter-strain virulence. We also identified another novel candidate T4E, ECHLIB_RS02720, a hypothetical protein exhibiting EPIYA, and NLS domains as well as a classical type IV secretion signal, suggesting an important role inside the host cell. Overall, our results agree with current knowledge of *Ehrlichia* molecular pathogenesis, and reveal novel candidate T4Es that require experimental validation. This work demonstrates that comparative effectomics enables identification of important host pathways targeted by the bacterial pathogen. Our study, which focuses on the type IV effector repertoires among several strains of *E. chaffeensis* species, is an original approach and provides rational putative targets for the design of alternative therapeutics against intracellular pathogens. The collection of putative effectors of *E. chaffeensis* described in our paper could serve as a roadmap for future studies of the function and evolution of effectors.

## Introduction

*Ehrlichia chaffeensis* is an intracellular rickettsial pathogen transmitted by *Amblyomma americanum* ticks, which is the etiologic agent of human monocytic ehrlichiosis (HME) (Dumler et al., 1993). This pathogen also causes disease in several other vertebrates, including dogs and deer (Paddock and Childs, 2003). The white-tailed deer is the reservoir host for *E. chaffeensis*, while humans, dogs and other vertebrate hosts, such as coyotes and goats, are regarded as incidental hosts (Paddock and Childs, 2003). This bacterium is able to replicate within two hosts, a mammalian host and a tick vector, and is capable of orchestrating highly sophisticated strategies to persist and infect their natural hosts (Rikihisa, 2010). Thus, studying *E. chaffeensis* provide a wealth of information about bacterial adaptation to various environments.

*E. chaffeensis* has a biphasic developmental cycle involving two morphologically distinct forms (Zhang et al., 2007). The infectious extracellular forms (dense core cells) first attach to the surface of host target cells before entering by endocytosis. Inside the host cells, the bacteria differentiate into reticulate cells within a membrane-bound vacuole where they create a safe niche for survival and replication by binary fission to form large colonies, called morulae. After a few days, the bacteria redifferentiate into infectious forms to be released outside the cell and start a new cycle of infection (Zhang et al., 2007).

In *E. chaffeensis*, the genome sequences of eight human isolates with variable pathogenicity, are available (Table 1). The first strain was discovered in 1991 in a 21-year old man and was named *Arkansas* for its geographic origin (Dawson et al., 1991). The most recently identified strain, called West Paces, was found in Tennessee in 2000 (Cheng et al., 2003). The other strains, Heartland, Jax, Liberty, Osceola, Saint Vincent, and Wakulla have also been isolated in humans (Table 1) and show different pathogenesis. In severe combined immunodeficiency (SCID) mice, Miura et al. observed differences in virulence in three of the strains, the Arkansas strain causing mild, the Liberty strain causing acute severe pathogenesis, and the Wakulla strain causing acute lethal pathogenesis (Miura and Rikihisa, 2007). The eight strains of *E. chaffeensis* used in this study were separated into three genetic groups based on the sequence polymorphisms of the *p28* outer membrane protein genes (Yu et al., 1999). The Arkansas and Osceola strains were classified in group I, the Heartland, Saint Vincent, and Wakulla strains in group II, and the Jax and Liberty strains in group III. The West Paces strain had not yet been isolated when the genetic groups were defined. Other genetic classifications were based on genes encoding immunoreactive proteins. The gene encoding tandem-repeat proteins (TRP) 32 (formerly VLPT, the variable length PCR target gene) contains the region specifying three to six nearly identical, highly hydrophilic 90–amino acid tandem repeats (Sumner et al., 1999). Similarly, in TRP120 (formerly gp120), there are two to four imperfect, direct, tandem 80 bp repeats (Sumner et al., 1999). The number of repeats varies depending on the isolate, resulting in variations in size in the encoded protein. The TRP32 gene shows great inter-strain diversity and is characterized by a series of direct tandem repeats whose number varies among isolates (Paddock and Childs, 2003). The DNA of TRP32 genes amplified from cultured isolates of *E. chaffeensis*, or from ticks, or from samples of patients' blood infected with this pathogen, has shown two to six repeats (summarized in Table 1). TRP120gene plays an important role in *E. chaffeensis* infection as it is a type I secretion system effector which is sumoylated on lysine residues and mediates interactions with host protein targets such as actin and myosin cytoskeleton components (Myo10) or GGA1 involved in vesicular trafficking (Wakeel et al., 2009) (Table 1).

**Table 1:** **Main biological and genetic characteristics of the eight *Ehrlichia chaffeensis* strains analyzed**.

**Strain**	**Arkansas**	**Heartland**	**Jax**	**Liberty**	**Osceola**	**St. Vincent**	**Wakulla**	**West Paces**
**Year**	1991	1999	1997	1998	1997	1996	1997	2000
**Origin**	Arkansas	Nebraska	Florida	Florida	Florida	Georgia	Florida	Tennessee
**Source**	21-year old male	Human	51-year old Woman	Human	Human	52-year old, HIV+	Human	Human
**Human, clinical and laboratory observations**	Fever, headache, pharyngitis, nausea, vomiting, and dehydration. Cervical lymphadenopathy, splenomegaly, thrombocytopenia, leukopenia with left shift, elevations in serum aspartate transaminase concentration.	HME, no clinical description available.	Fever, non-productive cough, nausea, vomiting, and diarrhea, profoundly lethargic. Thrombocytopenia, leukopenia, elevations in serum aspartate transaminase concentration, doxycycline therapy, cerebrospinal fluid mononuclear pleocytosis, pulmonary oedema, hypotension, and anuria. The patient died in hospital on day 6.	Acute HME, no clinical description available.	Acute HME, no clinical description available.	Fever, headache, myalgia, nausea, and vomiting, orthostatic hypotension, thrombocytopenia, leukopenia with left shift, elevations in serum aspartate transaminase concentration, doxycycline therapy. Lobar pneumonia and acute renal failure. The patient died.	Acute HME, no clinical description available.	Acute HME, no clinical description available.
**SCID mice pathogenesis**	Mild, chronic	UN	UN	Acute, severe	UN	UN	Acute, lethal	UN
**Genetic Group/ TRP32/TRP120 repeats**	I/4/4	II/3/3	III/4/4	III/4/4	I/4/3	II/3/3	II/6/4	II/3/3
**Literature**	Dawson et al., 1991	Sumner et al., 1999	Paddock et al., 1997	Sumner et al., 1999	Sumner et al., 1999	Paddock et al., 1997	Sumner et al., 1999	Cheng et al., 2003

*Human isolates of E. chaffeensis were classified in three genetic groups according to the 28-kDa major outer membrane gene cluster, the number of TRP32 repeats (variable-length PCR target gene, NCBI accession version # WP_011452439.1) and the number of TRP120 repeats (120-kDa immunodominant surface protein, NCBI accession version # WP_011452362.1). Data for pathogenesis in SCID mice for E. chaffeensis Arkansas, Liberty and Wakulla strains were obtained from Miura and Rikihisa (2007) and indicated. UN, unknown*.

Like other mammalian pathogenic bacteria, *E. chaffeensis* uses specific molecular mechanisms to evade host immune responses and to modulate host cell processes to its own advantage. Among these pathogenicity determinants, the type IV secretion system (T4SS) is a specialized protein complex involved in the injection of type IV effector (T4E) proteins into eukaryotic cells in order to subvert host cell processes during infection (Cascales and Christie, 2003). Rapid progress has been made toward identifying the proteins that form different parts of the T4SS, the translocated effectors and how these effectors subvert eukaryotic cellular processes during infection (Voth et al., 2012). However, to date, only two T4Es have been identified in the *Anaplasmataceae* family and shown to be critical for pathogenicity. After being injected in the host cells, AnkA (*Anaplasma phagocytophilum)*, is tyrosine-phosphorylated in the cytoplasm at EPIYA motifs and binds to SHP-1 phosphatase (Lin et al., 2007; Garcia-Garcia et al., 2009). AnkA is then translocated to the nucleus of the infected cell and interacts with gene promoter regions, leading to the downregulation of the CYBB and other key host defense genes (IJdo et al., 2007). In *E. chaffeensis*, the only known T4E is ECH_0825, homologous to *A. phagocytophilum* Ats-1 (Liu et al., 2012). This effector is translocated to host mitochondria where it restrains ROS and apoptosis for more efficient infection.

Our laboratory developed a searching algorithm for type IV effector proteins (S4TE), which identifies candidate T4Es in genome sequences based on a combinatorial approach with 14 different parameters (Meyer et al., 2013).

To better understand the evolution and pathogenicity of *E. chaffeensis*, we analyzed the eight available *E. chaffeensis* genomes of distinct geographical origin and of varying virulence isolated from humans (Table 1). We identified the relationship between *E. chaffeensis* strains using comparative whole genome analysis based on phylogenetic analysis, alignment of locally collinear blocks (LCB), and analysis of shared and specific genetic content. We provide evidence that the West Paces strain belongs to genetic group II and that *E. chaffeensis* is a highly conserved species. We describe likely virulence traits (candidate type IV effectors) encoded by their genomes and some putative host cell targets. Most notably some strains lack one or two candidate T4Es, but show conserved intracellular adaptive strategies.

Our results show that using our S4TE software and approach even for strains which are really close at the intraspecies level, enables the prediction of candidate type IV effectors that could be relevant for the study of bacterial pathogenesis.

## Materials and methods

### Retrieval of genome sequences and comparison of genomes

Complete genome sequences of *E. chaffeensis* strains were obtained from the National Center for Biotechnology Information (NCBI) database (ftp://ftp.ncbi.nih.gov/genomes/Bacteria/). Eight complete genomes were used in this study. Orthologous groups of all *E. chaffeensis* genes were identified using the PanOCT program (Fouts et al., 2012) with the following parameters: E-value 10^−5^, percent identity ≥ 30, and length of match ≥ 65.

### Prediction of *E. chaffeensis* type IV effectomes

The repertoires of T4Es were predicted using a S4TE algorithm with default parameters (Meyer et al., 2013). S4TE 1.4 predicts and ranks candidate T4Es by using a combination of 11 independent modules to explore 14 characteristics of type IV effectors. One module searches for consensus motifs in promoter regions; three modules search for the five features of the type IV secretion signal (C-terminal basicity, C-terminal charges, C-terminal hydrophobicity, overall hydrophilicity, and E-blocks); six modules search for several domains (eukaryotic–like domains, the DUF domain, EPIYA motifs, the nuclear localization signal, the mitochondrial localization signal, the prenylation domain, coiled-coil domains); and one module searches for homology with known T4Es (Meyer et al., 2013)

### Analysis of type IV effectome distribution according to local gene density

To visualize in a single representation the distance between each gene and its closest neighbors on the five prime and three prime borders, we sorted genes into two-dimensional bins defined by the length of their 5′ and 3′ flanking intergenic regions (hereafter denoted 5′ and 3′ FIRs) (Raffaele et al., 2010). The gene density distribution is represented in R by a heat map. We used the median length of FIRs to distinguish between gene-dense regions (GDRs); in-between regions (IBRs); and gene-sparse regions (GSRs). Putative type IV effectors identified by S4TE software were plotted on this graph according to their 5′ and 3′ FIRs (**Figure 2A**). The distribution of putative T4Es in each region was calculated for each strain (**Figure 2B**).

### Prediction of *E. chaffeensis* type IV effectors and host protein–protein interaction networks

Protein-protein interactions between human genomes and predicted type IV effectome of *E. chaffeensi*s were predicted using the Host-Pathogen Interaction Database (HPIDB) (Kumar and Nanduri, 2010) with the identity and percentage query coverage set at 30%. Based on the homology approach, the HPIDB predicts protein-protein interactions from a plentiful template of eukaryotic-prokaryotic inter-species interactions available among 68 hosts and 602 pathogens. Subcellular locations of the host proteins interacting with putative T4Es of *E. chaffeensis* were predicted using the CELLO2GO algorithm (Yu et al., 2014). S4TE 1.4 results were used to predict the location of T4Es (Meyer et al., 2013)

### Phylogenetic reconstruction and genomic plasticity analysis

For phylogenetic reconstruction, whole-genome nucleotide sequences of the eight *E. chaffeensis* strains were aligned using the progressiveMauve algorithm (Darling et al., 2010, http://gel.ahabs.wisc.edu/mauve/). FastTree was used with default parameters to build the unrooted tree (Price et al., 2009). Mauve software was also used to characterize the genomic rearrangements between the eight genomes of *E. chaffeensis* by showing LCBs. In order to accurately align conserved regions in the genomes, the progressiveMauve algorithm was parameterized with a match seed weight of 15 and a minimum LCB score of 70. The seed size parameter sets the minimum weight of the seed pattern used to generate local multiple alignments (matches) during the first pass of anchoring the alignment. The LCB weight sets the minimum number of matching nucleotides identified in a collinear region in order for the region to be considered a true homology rather than a random similarity (Darling et al., 2010).

## Results

### The *Ehrlichia chaffeensis* genomes are highly conserved

**Figure 1:**
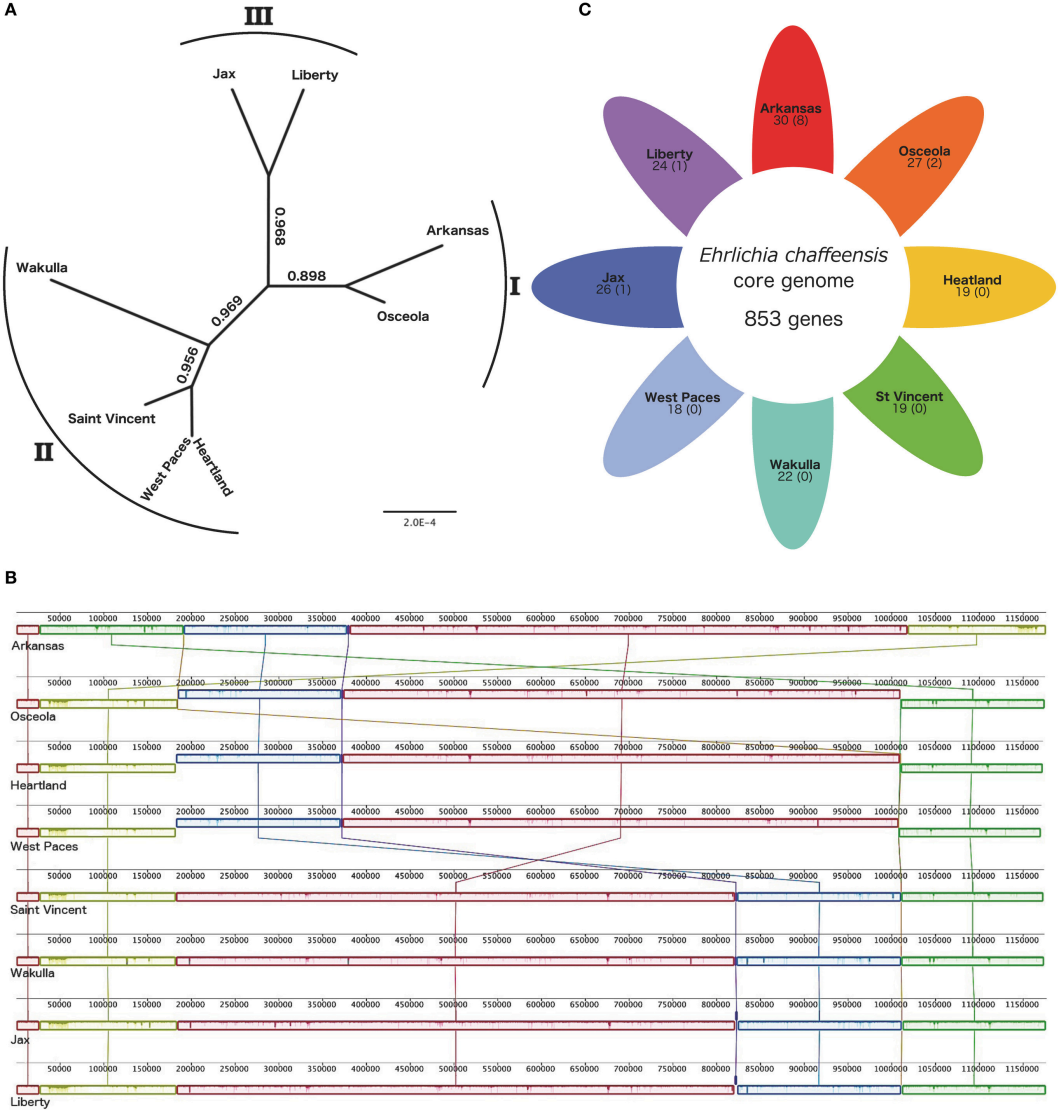
**Comparative genomics of 8 *Ehrlichia chaffeensis* strains. (A)** Phylogenetic tree of 8 *E. chaffeensis* strains. FastTree based on the Mauve alignment of the whole genomes of 8 *E. chaffeensis* strains. The node values indicate the local support values of the Shimodaira-Hasegawa test. The number outside the tree shows the genetic group of each strain, the West Paces strain was assigned to genetic group II due to the high level of conservation with the Heartland strain. **(B)** Alignments of 8 *E. chaffeensis* genomes generated using Mauve software (Darling et al., 2010) (http://gel.ahabs.wisc.edu/mauve/). Locally collinear blocks (LCBs), shown as rounded rectangles, represent regions with no rearrangement of homologous sequences across genomes. The forward or reverse orientation of the LCBs is indicated by their position, respectively above or below the line. Lines between the genomes trace orthologous LCBs. Using default parameters resulting in a minimum LCB weight of 70, there are 7 LCBs across all the genomes. The LCB weight defines the minimum number of matching nucleotides in a collinear region for it to be considered homologous across genomes and not the result of a spurious match. Regions outside LCBs were too divergent in at least one genome to be aligned successfully. Inside each LCB, vertical bars represent the similarity profile of the genome sequence. The height of each bar corresponds to the average level of conservation in that region of the genome sequence. **(C)** Shared and specific gene content between 8 *E. chaffeensis* strains. Each colored petal represents a different *E. chaffeensis* genome. The number in the center of the diagram represents the number of orthologous genes shared by all the genomes, thus defining the *E. chaffeensis* core genome. The number inside each individual petal corresponds to the number of genes that are absent from the core genome, and the numbers in brackets correspond to the number of genes specific to this strain. The number outside each petal shows the genetic group of each strain.

In order to establish a whole genome-based phylogeny of these eight *E. chaffeensis* strains, we used the Mauve progressive alignment and FastTree to build the tree. Our results are in agreement with those of previous studies, with the eight strains being separated into three genetic groups. The Arkansas and Osceola strains were assigned to group I, and the Wakulla, Saint Vincent, West Paces, and Heartland strains were assigned to group II. We also assigned the West Paces strain to group II due to its phylogenetically close proximity to the Heartland strain (Figure 1A). The Jax and Liberty strains were assigned to group III (Figure 1A). With an average size of 1.2 Mb, the genomic features of the eight strains used in this study are similar. The GC (guanine-cytosine) content was seen to be highly homogenous (30.1%) and genome sequences relatively well-conserved (Figure 1B). The number of genes ranges from 871 to 883. Whole genome alignments revealed seven LCBs with some inversions and with rearrangements in the genomes with respect to one another (Figure 1B). In the Arkansas strain, we found a rearrangement between three LCBs with green and orange blocks switched with yellow LCB. The strains Arkansas, Osceola, Heartland, and West Paces showed an inversion of blue and red LCBs compared to other genomes. The Saint Vincent and Wakulla strains showed inversion of one small LCB (purple, Figure 1B). The structural variation among these genomes suggests a low degree of inter-species genome plasticity for *E. chaffeensis*.

We then analyzed the pan-genome of *E. chaffeensis*. We used PanOCT software to cluster the ortholog and compared the core and accessory genomes of the eight strains of *E. chaffeensis*. (Figure 1C). The *E. chaffeensis* core-genome contained 853 orthologous genes, corresponding to ~96% of the pan-genome and indicating that the *E. chaffeensis* accessory genome is narrow. Thus, four percent of *E. chaffeensis* genes are not in the core genome and only a few genes are specific to four out of these eight strains. The Arkansas strain harbored eight specific genes, the Osceola strain two specific genes and Liberty and Jax strains only one specific gene (Figure 1C).

To test if the genome plasticity and effector repertoires can explain the differential intra-species pathogenesis of *E. chaffeensis*, we decided to focus our study on four different representative strains. When data were available, we chose strains belonging to different genetic groups showing variations in virulence. From genetic group I, we chose the Arkansas strain, which is the most widely studied and best-described strain in the literature. This strain shows mild virulence in immunodeficient mice (Miura and Rikihisa, 2007). From genetic group II, we chose the West Paces and Wakulla strains, the latter causing acute lethal pathogenesis in SCID mice (Miura and Rikihisa, 2007). Finally, from genetic group III, we chose the Liberty strain, which causes acute pathogenesis in immunodeficient mice (Miura and Rikihisa, 2007).

### Prediction of type IV effectors for *E. chaffeensis* identifies the core type IV effectome among four human isolates

We used the S4TE algorithm to predict and compare the type IV effector repertoires in four human isolates (Arkansas, Liberty, Wakulla, and West Paces) of *E. chaffeensis* in order to determine how these repertoires differed between strains with respect to the presence or absence of whole candidate T4Es. We identified a conserved repertoire of 45 candidate T4Es, defining the core type IV effectome of *E. chaffeensis*.

Based on orthology analysis, we found few differences in T4E content between the four selected *E. chaffeensis* isolates. *E. chaffeensis* str. Liberty was the only strain to own all 47 predicted T4Es (Table 2). One candidate T4E, ECHLIB_RS02720, is specific to *E. chaffeensis* str. Liberty, whereas ECHLIB_RS04640 was only absent in *E. chaffeensis* str. West Paces. All the other predicted T4Es (94% of predicted effectors) are common to the four strains, revealing the low diversity of effector repertoires in *E. chaffeensis* species. We did not discover any relation between the presence or absence of an effector and the variations in virulence exhibited by the Wakulla, Liberty, and Arkansas strains.

**Table 2:** **Putative type IV effectors (T4Es) identified by the S4TE algorithm**.

***Ehrlichia chaffeensis* strains**				**NCBI protein names**																
**Arkansas**	**Liberty**	**Wakulla**	**West Paces**		**S4TE Score**	**Promoter motif**	**Homology**	**Euk-like domain**	**EPIYA**	**NLS**	**MLS**	**Prenylation**	**Coiled coils**	**Cter basicity**	**Cter charges**	**Cter basicity**	**Cter charges**	**Cterhydrophobicity**	**Eblock**	**Location**
ECH_RS02870	ECHLIB_RS01940	ECHWAK_RS01950	ECHWP_RS02750	Hypothetical protein	229	1	1	1	0	1	1	0	0	0	1	0	0	1	0	GSRs
ECH_RS03425	ECHLIB_RS01385	ECHWAK_RS01390	ECHWP RS03295	Hypothetical protein	164	1	1	0	0	0	0	0	0	0	0	1	0	0	0	GDRs
ECH_RS04335	ECHLIB_RS00490	ECHWAK_RS00485	ECHWP_RS00485	Gamma carbonic anhydrase family protein	151	1	1	0	0	0	0	0	0	0	1	0	0	0	1	GSRs
ECH_RS02750	ECHLIB_RS02060	ECHWAK_R502070	ECHWP R502630	Hypothetical protein	141	1	0	1	0	1	1	0	0	0	1	0	0	0	0	GDRs
ECH_RS02620	ECHLIB_RS02190	ECHWAK_RS02200	ECHWP_RS02500	Alpha/beta hydrolase	139	1	1	0	0	0	1	0	0	0	1	1	0	0	0	GSRs
ECH_RS03745	ECHLIB_RS01065	ECHWAK_RS01070	ECHWP RS03615	AI-2E family transporter	122	1	1	0	0	0	0	0	0	0	1	0	0	0	0	GSRs
ECH_RS00450	ECHLIB_RS04345	ECHWAK_RS04360	ECHWP_RS04320	Hypothetica l protein	118	1	0	0	1	0	1	0	0	0	1	0	0	0	1	GSRs
ECH_RS01210	ECHLIB_RS03585	ECHWAK_RS03600	ECHWP RS01105	DNA ligase (NAD(+)) LlgA	117	0	0	1	0	1	1	0	0	0	1	0	0	1	0	BRs
ECH RS04225	ECHLIB RS00595	ECHWAK RS00600	ECHWP RS00590	Hypothetica l protein	115	1	0	0	0	1	1	0	0	0	1	1	0	1	0	GSRs
	ECHLIB_RS02720			Hypothetical protein	114	0	0	0	0	1	1	0	0	0	1	0	0	1	1	GDRs
ECH_RS02365	ECHLIB_RS02435	ECHWAK_RS02455	ECHWP_RS02250	Translation initiation factor IF-2	114	0	0	0	0	1	1	0	0	0	0	1	0	1	1	GDRs
ECH_RS03205	ECHLIB_RS01605	ECHWAK_RS01615	ECHWP RS03075	Diguanylate cyclase response regulator0	109	0	1	0	0	0	1	0	0	0	1	0	0	0	0	IBRs
ECH_RS03195	ECHLIB_RS01615	ECHWAK_RS01625	ECHWP_RS03065	NAD-glutamate dehydrogenase	108	1	0	0	0	1	0	0	1	0	1	0	1	1	0	IBRs
ECH_RS02495	ECHLIB_RS02315	ECHWAK_R502330	ECHWP R502375	Peptide chain release factor 1	105	1	0	0	0	0	0	0	0	0	1	0	0	1	1	IBRs
ECH_RS04685	ECHLIB_RS04640	ECHWAK_RS04650		Hypothetica l protein	103	0	0	0	1	1	1	0	0	0	1	1	1	1	0	GSRs
ECH_RS01570	ECHLIB_RS03225	ECHWAK_RS03240	ECHWP RS01465	Hypothetical protein	101	0	0	0	0	1	1	0	0	0	1	1	1	1	0	GDRs
ECH_RS04230	ECHLIB_RS00590	ECHWAK_RS00595	ECHWP_RS00585	Hypothetica l protein	99	1	0	0	0	1	1	0	0	0	0	0	0	1	0	GSRs
ECH RS02945	ECHLIB RS01860	ECHWAK RS01875	ECHWP RS02825	Transcriptional regulator	98	0	1	0	0	0	0	0	0	0	1	0	0	0	0	IBRs
ECH_RS02385	ECHLIB_RS02415	ECHWAK_RS02435	ECHWP_RS02270	Hypothetica l protein	98	1	0	0	0	1	0	0	0	0	1	0	0	1	0	GSRs
ECH_RS03860	ECHLIB_RS00950	ECHWAK_RS00955	ECHWP RS03725	Hypothetical protein	97	1	0	0	0	0	1	0	0	0	1	0	1	1	0	IBRs
ECH_RS04650	ECHLIB_RS00175	ECHWAK_RS00175	ECHWP_RS00175	Protein translocase subunit SecA	93	1	0	0	0	0	1	0	0	0	1	1	0	1	0	GSRs
ECH_RS02080	ECHLIB_R502725	ECHWAK_R502740	ECHWP R501965	Hypothetical protein	93	0	0	0	0	1	0	0	0	0	0	0	0	0	1	GDRs
ECH_RS02075	ECHLIB_RS02730	ECHWAK_RS02745	ECHWP_RS01960	Conjugal transfer protein Trbl	92	0	0	0	0	0	1	0	0	0	1	0	0	1	1	GDRs
ECH_RS03040	ECHLIB_RS01765	ECHWAK_R501780	ECHWP R502910	Peptidylprolyl isomerase	91	0	0	0	0	1	1	0	0	0	1	1	0	1	0	GSRs
ECH_RS02255	ECHLIB_RS02545	ECHWAK_RS02565	ECHWP_RS02140	165 rRNA (uracii(1498)-N(3)) -methyltransferase	88	0	1	0	0	0	0	0	0	0	0	0	0	0	0	GDRs
ECH_RS03605	ECHLIB_RS01205	ECHWAK_R501210	ECHWP R503475	Hypothetical protein	87	1	0	1	0	0	1	0	0	0	1	0	1	0	0	IBRs
ECH RS03515	ECHLIB RS01295	ECHWAK RS01300	ECHWP RS03385	Hypothetical protein	87	1	0	0	0	0	1	0	0	0	1	0	0	1	0	GSRs
ECH_RS02340	ECHLIB_RS02460	ECHWAK_RS02480	ECHWP RS02225	Hypothetical protein	87	1	0	0	0	0	1	0	0	0	1	0	0	1	0	IBRs
ECH_RS01565	ECHLIB_RS03230	ECHWAK_RS03245	ECHWP_RS01460	Exodeoxyribonuclease V subunit beta	87	1	0	0	0	0	1	0	0	0	1	0	0	1	0	IBRs
ECH_RS01140	ECHLIB_RS03660	ECHWAK_RS03675	ECHWP RS01035	Hypothetical protein	87	1	0	0	0	0	1	0	0	0	1	0	0	1	0	GSRs
ECH_RS03890	ECHLIB_RS00920	ECHWAK_RS00925	ECHWP_RS03755	DNA-directed RNA polymerase subunit beta	85	0	0	0	0	1	1	0	0	0	1	0	0	1	0	GDRs
ECH_RS00205	ECHLIB_RS04580	ECHWAK_RS04590	ECHWP RS04555	Type IV secretion system protein VirD4	85	0	0	0	0	1	1	0	0	0	1	0	0	1	0	IBRs
ECH_RS03630	ECHLIB_RS01180	ECHWAK_RS01185	ECHWP_RS03500	DNA processing protein DprA	84	1	0	0	0	0	1	0	0	0	1	0	1	0	1	GSRs
ECH_RS03440	ECHLIB_RS01370	ECHWAK_RS01375	ECHWP RS03310	Phage capsid protein	82	1	0	0	0	0	0	0	0	0	1	1	0	1	0	GSRs
ECH_RS03530	ECHLIB_RS01280	ECHWAK_RS01285	ECHWP_RS03400	Hypothetica l protein	81	0	0	0	0	0	0	0	0	0	1	0	0	1	1	IBRs
~RS01950	ECHLIB_RS02855	ECHWAK_RS02870	ECHWP RS01835	Molecular chaperone DnaK	81	0	0	0	0	0	0	0	0	0	1	0	0	1	1	IBRs
ECH_RS03610	ECHLIB_RS01200	ECHWAK_RS01205	ECHWP_RS03480	Hypothetica l protein	80	0	0	0	0	1	0	0	0	0	1	1	0	1	0	GSRs
ECH_RS03260	ECHLIB_RS01550	ECHWAK_RS01560	ECHWP RS03130	abc-ATPase UvrA	77	1	0	0	0	0	1	0	0	0	0	0	0	1	0	GSRs
ECH_RS03895	ECHLIB_RS00915	ECHWAK_RS00920	ECHWP_RS03760	505 ribosomal protein L7 /L12	74	1	0	0	0	0	1	0	0	0	1	0	0	0	1	GDRs
ECH_RS02525	ECHLIB_RS02285	ECHWAK_RS02300	ECHWP RS02405	Glutamate-tRNA ligase	74	1	0	0	0	0	1	0	0	0	1	0	0	0	1	GSRs
ECH RS00785	ECHLIB_RS04010	ECHWAK_RS04025	ECHWP_RS03985	Hypothetica l protein	74	0	0	0	0	1	0	0	0	0	1	0	0	1	0	GSRs
'Eei(RS00330	ECHLIB_RS04455	ECHWAK_RS04465	ECHWP RS04430	1-acyl-sn-glycerol-3-phosphate acyltransferase	74	1	0	0	0	0	1	0	0	0	1	0	0	0	1	GSRs
ECH_RS00255	ECHLIB_RS04530	ECHWAK_RS04540	ECHWP_RS04505	Hypothetical protein	74	1	0	0	0	0	1	0	0	0	1	0	0	0	1	BRs
ECH_RS03415	ECHLIB_RS01395	ECHWAK_RS01400	ECHWP RS03285	NAD+ synthetase	73	1	0	0	0	1	1	0	0	0	1	1	0	0	0	GSRs
ECH_RS02490	ECHLIB_RS02320	ECHWAK_RS02335	ECHWP_RS02370	GTP-binding protein	73	1	0	0	0	0	0	0	0	0	1	0	1	0	1	IBRs
ECH_RS00505	ECHLIB_RS04290	ECHWAK_RS04305	ECHWP_RS04265	Citrate (Si)-synthase	73	1	0	0	0	0	0	0	0	0	0	1	0	1	1	GDRs
ECH RS03555	ECHLIB RS01255	ECHWAK RS01260	ECHWP RS03425	Hypothetica l protein	72	0	0	0	0	0	0	0	1	0	0	1	1	0	1	GSRs

*This table shows the candidate T4Es identified by S4TE software in four E. chaffeensis strains. The Liberty strain is used as a reference to sort predicted effectors, and the homolog candidate effectors are ranked by S4TE scores. Each T4E is defined by the gene ID, Name, and S4TE features*.

Identified candidate T4Es were sorted according to their S4TE score, which ranged from 72 (corresponding to the S4TE algorithm threshold) to 229 (Table 2). Eight candidate T4Es showed homology with known T4Es (17% of predicted T4Es) as indicated by the number “1” in the Homology column in Table 2. Among these candidates, one is ECH_RS01385 previously called ECH0825 (old NCBI locus_tag) (Liu et al., 2012; Table S1). This effector was predicted with the second highest S4TE score of 164. The first predicted T4E, ECH_RS01940, matched the homologous gene of *A. phagocytophilum* AnkA (IJdo et al., 2007; Lin et al., 2007; Garcia-Garcia et al., 2009; Table S1). ECHLIB_RS02190, ECHLIB_RS01065, ECHLIB_RS01605, and ECHLIB_RS01860 are four candidate T4Es presenting homologies with known *Coxiella burnetii* effectors (Table S1). ECHLIB_RS022545 shows homology with a known *Legionella pneumophila* T4E (lpg2936, 16S ribosomal RNA methyltransferase RsmE) while ECHLIB_RS00490 shows homology with a *Brucella* effector (Table S1).

Besides homology with known effectors, several other candidate T4Es had interesting features (Table 2). Indeed, 59.6% of predicted T4Es had a promoter motif such as PmrA upstream of the effector genes of *Coxiella* spp. and *Legionella* spp. Furthermore, 8.5% of putative T4Es harbored eukaryotic-like domains such as AnkA (Ankyrin repeat-containing domain) and BRCT (phospho-protein binding domain) domains. Only two putative T4Es contained domains of unknown function (DUF). It is interesting to note that 38.3 and 61.7% of candidate T4Es had a tyrosine phosphorylation domain (EPIYA) and a nuclear localization signal (NLS), respectively. Moreover, nearly 38% of the proteins harboring an NLS also had an EPIYA phosphorylation domain. None of the predicted T4Es had a prenylation domain or a coiled-coil domain. Thirty-four percent of the candidate T4Es harbored the canonical *L. pneumophila* secretion domain (E-block).

Concerning other features related to the type IV secretion signal, 17% of the predicted T4Es showed C-terminal hydrophobicity, 68% showed global hydropathy < −200 (on the Kyte-Doolittle scale), 21.3% had a C-ter charge ≥ 2 and 89.4% had at least three alkaline amino acids in C-terminal 25 amino acids.

### Putative type IV effectors of *E. chaffeensis* are overrepresented in gene sparse regions of the genome

**Figure 2:**
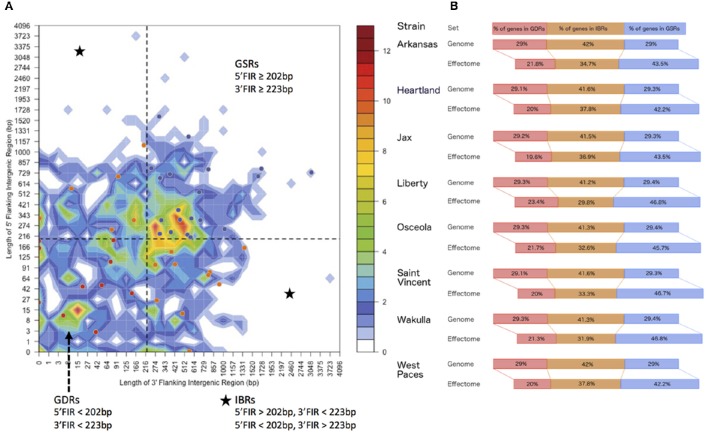
**Distribution of *Ehrlichia chaffeensis* effectomes according to local gene density. (A)** Distribution of *E. chaffeensis* str. Arkansas genes according to the length of their flanking intergenic regions (FIRs). All *E. chaffeensis* genes were sorted into 2-dimensional bins according to the length of their 5′ (y-axis) and 3′ (x-axis) FIRs. The number of genes in the bins is represented by a color-coded density graph. Genes whose FIRs are both longer than the median FIR length were considered as gene-sparse region (GSR) genes. Genes whose FIRs are both below the median value were considered as gene-dense region (GDR) genes. In-between region (IBR) genes are genes with a long 5′FIR and short 3′FIR, or inversely. Candidate effectors predicted using the S4TE algorithm were *s* plotted on this distribution according to their own 3′ and 5′ FIRs. A color is assigned to each of the three following groups: Red to GDRs, orange to IBRs, and blue to GSRs. **(B)** Distribution of genes in GDRs, IBRs, and GSRs of *E. chaffeensis* strains. The proportion of the genome and the effectome that occurs in GDRs (red), IBRs (orange), and in GSRs (blue) is indicated.

In order to understand how genomic plasticity influences the distribution of predicted T4Es, we first analyzed the genome architecture of *E. chaffeensis* by looking at local gene density (Figure 2). The median length of 3′ and 5′ flanking intergenic regions (FIRs) delimits four coherent gene pools when combined with the 2-variable binning representations (Figure 2A).

The gene dense region (GDR, genes with 5′ FIR < 202 bp and 3′ FIR < 223 bp) contains 254 genes, which account for 28.9% of the *E. chaffeensis* str. Arkansas genome (Figure 2A). The gene sparse region (GSR, genes with 5′ FIR ≥ 202 bp and 3′ FIR ≥ 203 bp) includes 255 genes, which account for 29% of the genome (Figure 2A).

The other two quadrants define in-between regions (IBRs) grouping genes with a 5′ FIR shorter than the median length and a longer 5′ FIR, and inversely. In the *E. chaffeensis* str. Arkansas genome, 370 genes, which account for 42% of the genome, fall into IBRs (Figure 2A). This genome architecture of *E. chaffeensis* str. Arkansas is representative of other strains of *E. chaffeensis* (Figure 2B).

We then performed a detailed analysis of the distribution of predicted *E. chaffeensis* T4Es according to local gene density. We found that the predicted T4Es of all isolates of *E. chaffeensis* frequently had both FIRs above the genome median value. Although 29% of *E. chaffeensis* genes belong to GSRs, 42.2% to 46.8% of predicted type IV effector genes fall in GSRs (Figures 2A,B). Thus, compared to the whole genome, the GSRs showed a 1.5-fold enrichment in candidate type IV effector genes. Consequently, the proportion of candidate T4Es in the GDRs and IBRs is lower than the proportion of genes of the whole genome (Figure 2B). These results suggest that plastic regions with low gene density harbor pathogenicity genes and could play a role in host-bacteria interactions.

### Prediction of the host-pathogen protein–protein interaction network

**Figure 3:**
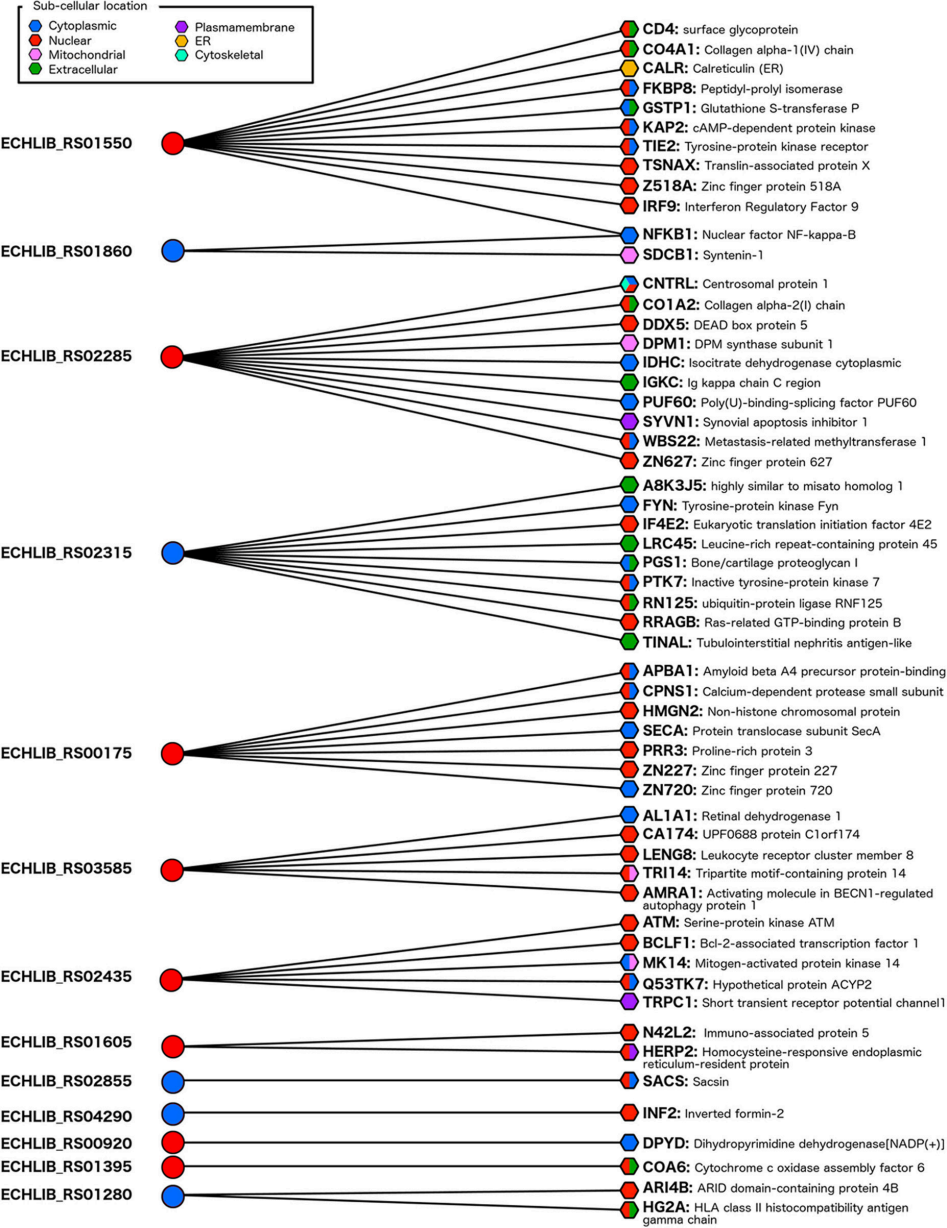
**Protein-protein interaction network between the *E. chaffeensis* str. Liberty effectome and the human genome**. A sub-cellular location was predicted with the S4TE algorithm (http://sate.cirad.fr) for *Ehrlichia* candidate effectors (left) and with CELLO2GO software (http://cello.life.nctu.edu.tw/cello2go/) for human proteins (right). Blue and red circles represent predicted T4Es located in the cytoplasm and in the nucleus of the host cell, respectively. Blue, red, pink, green, purple, yellow, and turquoise hexagons represent the different locations of targeted human proteins in the host cell. Hexagons harbor several colors when CELLO2GO predicts several probable subcellular locations.

We predicted the interactions of *E. chaffeensis* T4Es with human proteome and identified 57 protein-protein interactions with the involvement of 13 putative T4Es of *E. chaffeensis* str. Liberty (which harbors all predicted T4Es) and 56 human proteins (Figure 3).

The targeted host proteins are located in cellular compartments relevant to the pathogenesis mechanisms. The predicted cellular localizations of human interacting proteins were confirmed in cytoplasm, nucleus, extracellular, mitochondrial, plasma membrane, endoplasmic reticulum, and cytoskeleton (Figure 3). As described above, we predicted the subcellular localization in human host cell of *E. chaffeensis* T4Es using the S4TE algorithm (Table 2, Table S1). Out of the 13 predicted T4Es of *E. chaffeensis* that interact with human proteins, eight (~60%) harbor at least one nuclear location signal (NLS). Interestingly, most of these proteins had putative human targets located in the nucleus (Figure 3).

Thus, the putative targets of the ABC-ATPase UvrA (ECHLIB_RS01550) are involved in different processes including innate immunity, response to stress, the cell cycle, cell signaling, and cell death (Table S2). Another candidate nuclear effector (ECHLIB_RS02285) interacts with 11 human proteins, most of which are involved in metabolic processes such as amino acid synthesis (IDHC), carbohydrate metabolic process (DPM1 and IDHC), lipid metabolism (DPM1), and nitrogen compound metabolism (ZN627, IDHC, DPM1, WBS22, CNTRL, and PUF60) (Table S2).

The nuclear effectors ECHLIB_RS02315 and the DNA ligase ECHLIB_RS03585 interact with several putative targets involved in immune and stress responses, cell organization, and cell death. Most of the proteins targeted by ECHLIB_RS00175 are located in the nucleus and are involved in nuclear organization (chromosomal protein HMG2) or biosynthetic process (proline-rich and zinc finger proteins) (Table S2). The nuclear effector ECHLIB_RS02435 interacts with kinases and with the nuclear transcriptional repressor BCLF1 suggesting an important role in signal transduction and stress response, particularly activation of response to DNA damage. It is of note that this effector also harbors a tyrosine phosphorylation domain that could play an important role in the ATM/MAP kinases signaling pathway.

A dihydropyrimidine dehydrogenase is the only target of ECHLIB_RS00920 involved in catabolic and metabolic processes. The last putative nuclear effector with a target is a ligase (ECHLIB_RS01395), which interacted with a protein associated with cytochrome c oxidase and had one putative target on the human genome. This protein plays a role in the organization of mitochondria, the assembly of cell components and in the generation of precursor metabolite and energy.

Among the other *E. chaffeensis* T4Es whose interaction with human proteins was predicted, the transcriptional regulator ECHLIB_RS01860 interacts with two putative targets. The first is the nuclear factor NF-kappa-B, which plays a prominent role in immune responses, responses to stress, and cell death. The second target is SDCB1, which is involved in cytoskeleton organization, cell-cell signaling, locomotion, cell adhesion, and growth (Table S2).

Finally, four other cytoplasmic *E. chaffeensis* T4Es (ECHLIB_RS01605, ECHLIB_RS02855, ECHLIB_RS04290, ECHLIB_RS01280) were predicted to interact with one or two proteins involved in reticulum catabolic processes (HERP2), protein folding (SACS), cytoskeleton organization (INF2) and transcriptional repression (ARI4B), or in immune response (antigen processing by HG2A), respectively.

## Discussion

Motivated by the availability of eight genome sequences, we explored the world of pathogenicity determinants in the species *E. chaffeensis*. We hypothesized that variations in virulence between some strains could be driven by genome plasticity and the acquisition of different repertoires of type IV effectors (T4Es). Such mechanisms of evolution have already been observed in plant pathogenic and non-pathogenic *Xanthomonas* (Cesbron et al., 2015). The aim of our work was to show that computational methods to identify and categorize putative T4Es, prior to their functional characterization, could be a valuable approach to better understand *E. chaffeensis*-host interactions. We also aimed to identify novel candidate T4Es and their interactions with host cell proteins to advance our current understanding of *E. chaffeensis* pathogenesis.

We showed that *E. chaffeensis* genomes had low plasticity and with few intra-species genomic rearrangements. We also showed that the eight genomes of *E. chaffeensis* are highly conserved with 96% genes present in the core genome. Hence, the observed differences in pathogenesis and symptoms between the Arkansas, Liberty and Wakulla strains (Table 1) could be due to the absence of certain genes in the core genome.

The core type IV effectome of a bacterial species is defined by the minimum set of type IV effectors conserved in all strains within a species, which make it necessary for the bacterium to develop inside the host cell. Using our comparative genomics approach, we showed that the core type IV effectome of *E. chaffeensis* contains 45 candidate T4Es. In addition, we showed that the Liberty isolate of *E. chaffeensis* contains all the 47 predicted T4Es. Although, S4TE software was designed for optimal sensitivity (Meyer et al., 2013), the prediction of false positives can occur and is inherent to any predictive computational approach.

However in our study, the S4TE algorithm correctly predicted the two known type IV effectors in *Anaplasmataceae* family with *E. chaffeensis* mitochondrial effector ECH0825 (ECHLIB_RS01385) and the homolog of *A. phagocytophilum* nucleomodulin AnkA (ECHLIB_RS01940) (Table S1). In addition, S4TE predicted effectors that are homologous to known effectors in other bacteria, including *C. burnetii, L. pneumophila*, and *Brucella* spp. S4TE also predicted some new candidate T4Es that were not easy to identify *ab initio*, based solely on the poor quality of automated genome annotations, especially for bacteria harboring 30% or more unknown hypothetical proteins like *Anaplasmataceae*. For example, S4TE identified some bacterial enzymes as candidate effectors, including the annotated acyltransferase ECHLIB_RS04455, which is in agreement with current knowledge on bacterial effectors (Anderson et al., 2015).

Most of the predicted T4Es in *E. chaffeensis* belong to the core type IV effectome, showing that effector repertoires are highly conserved in this species. Thus, for bacteria with compact genomes, the type IV effector repertoires may not reflect the genetic diversity and the variations in pathogenesis observed within a species. However, two candidate T4Es, ECHLIB_RS02720 and ECHLIB_RS04640, are not present in all *E. chaffeensis* strains and could explain some within-strain variations in virulence. Indeed, in pathogens with bigger genomes and more complex lifestyles, some authors demonstrated that diversity in effector repertoires is linked to host specificity (Cooke et al., 2012; Guyon et al., 2014; Schwartz et al., 2015). The 45 T4Es predicted by S4TE in *E. chaffeensis* account for about 5% of the genome. In comparison, in the facultative intracellular *L. pneumophila* str. Philadelphia I, which contains a well-characterized type IV effectome, 286 T4Es account for about 9% of the genome (Lifshitz et al., 2013). Thus, in relation to the number of genes, the predicted type IV effectome of *E. chaffeensis* is significantly smaller than that of *L. pneumophila*. This could be explained by the reduced size of the *E. chaffeensis* genome, linked to its obligate intracellular lifestyle, thus leading to less functional redundancy in the type IV effectome.

Interestingly, the *E. chaffeensis* Liberty strain contained one specific candidate T4E, ECHLIB_RS02720, a hypothetical protein exhibiting EPIYA and NLS domains as well as a classical type IV secretion signal. These features strongly suggest this effector could be phosphorylated in the cytoplasm, addressed to the nucleus, and play an important role inside the host cell, like the AnkA effector of *A. phagocytophilum* (IJdo et al., 2007; Garcia-Garcia et al., 2009). This effector could also be involved in the differential virulence phenotypes described between the Arkansas and Liberty strains in SCID mice (Miura and Rikihisa, 2007). Conversely, the identical putative type IV effectomes of the Arkansas and Wakulla strains cannot explain their differential pathogenesis in SCID mice. We cannot exclude the possibility that the homologous T4Es repertoires of these two strains contain point mutations in some effectors, which would alter the pathogenesis of the strain, as shown in *L. pneumophila* with the mutant protein kinase LegK2 (Hervet et al., 2011). Another explanation could be differences in the metabolisms or the kinetics of infection of the Arkansas and Wakulla strains. Indeed, Marcelino et al. showed that virulent and attenuated Gardel strains of *E. ruminantium*, which have the same gene content, only differ in their proteome expression, yet have different life cycles (Marcelino et al., 2015). At the whole genome level, some horizontal gene transfer (HGT) of genes that control advantageous phenotypic differences, might also have occurred during evolution to explain the differing degrees of virulence between Wakulla, Liberty and Arkansas isolates of *E. chaffeensis* (Dorman et al., 2016).

We demonstrated that predicted T4Es are preferentially distributed in gene sparse regions of the genome. In addition, some putative effectors harbor typical eukaryotic features such as Ank or BRCT domains. These results suggest that some effectors could be acquired via HGT from other bacterial species (McAdam et al., 2014) or from the host cell (Lurie-Weinberger et al., 2010).

To guide the functional characterization of the candidate T4Es of interest with respect to *E. chaffeensis* pathogenesis, we tried to predict some putative host targets. Among the 47 candidate T4Es in *E. chaffeensis* str. Liberty, most of the proteins with predicted NLSs were predicted to interact with human proteins located in the nucleus. Moreover, several putative targets of candidate T4Es affect human immunity-related proteins. Two predicted T4Es (ECHLIB_RS01550 and ECHLIB_RS01860) could interact with the nuclear factor NF-kappa-B1. This is a pleiotropic transcription factor induced by a vast array of stimuli and which is linked to many biological processes, including immunity, inflammation, and apoptosis. Another predicted T4E (ECHLIB_RS01280) may play a role in controlling innate immune responses by interacting with two human proteins in particular, ARIA4B and HG2A. The first is a transcriptional repressor, and the second plays a critical role in MHC class II antigen processing by stabilizing peptide-free class II alpha/beta heterodimers in a complex. Suppressing innate immunity of the host cells is one of the necessary actions for the proper development of this intracellular bacterium (Luo, 2012).

Other putative T4Es could affect host cell transcription like ECHLIB_RS01605, which targets two transcriptional repressors: N42L2 and HERP2. On the other hand, some putative targets involve the global organization of cell membranes. Thus, COA6 is involved in the maturation of the mitochondrial respiratory chain complex IV; CO1A2 and CO4A1 are involved in the extracellular membrane by forming fibrillar collagen, with SDCB1 playing a role in vesicular trafficking (Zimmermann et al., 2001). This modification of global membrane organization could be related to the lysosome-like vacuole recruitment in intracellular bacteria, as shown in *C. burnetii* (Moffatt et al., 2015).

Our analysis of the protein-protein interaction network also revealed that certain candidate T4Es could alter the phosphorylation cascades by putatively interacting with protein kinases (FYN, PTK7, TIE2, KAP2, ATM, MK14), enzymes which catalyze phosphorylation reactions (Dhanasekaran and Premkumar Reddy, 1998). Phosphorylation-dephosphorylation mechanisms are extremely common in signaling pathways where they regulate cell activity (Dhanasekaran and Premkumar Reddy, 1998). For example, PTK7 is a catalytically inactive receptor tyrosine kinase which is upregulated in many common human cancers. Knocking down this protein was shown to inhibit cell proliferation and induce apoptosis (Meng et al., 2010). MK14 is a serine/threonine kinase, which is an essential component of the MAP kinase signaling pathway. MK14 is one of the four p38 MAPKs that play important roles in the cascade of cell responses induced by extracellular stimuli, such as pro-inflammatory cytokines or physical stress, leading to direct activation of transcription factors (Lo et al., 2014). Blocking these cascades could enable the bacterium to evade the innate immune response of the host cell. ATM/MKA14 regulatory networks have also been shown to regulate cytoplasmic targets, resulting in extensive cytoskeletal rearrangements (Pines et al., 2011). Acting on these cascades could favor the maturation of *Ehrlichia*-containing vacuoles, as shown for *L. pneumophila* which controls vesicle trafficking to escape host defenses and counteract the endocytic pathway (Michard et al., 2015). Finally, some candidate T4Es could affect metabolic proteins, like SYVN1, which acts as an E3 ubiquitin-protein ligase. Ubiquitination is a post-traductional biochemical modification that mainly leads to the degradation of ubiquitinated proteins by the proteasome. Moreover, it has been shown that ubiquitination of proteins in the endoplasmic reticulum negatively regulates the stress-induced apoptotic signaling pathway (Kaneko et al., 2002). Interestingly, we found another candidate T4E predicted to interact with the SACSIN molecular chaperone, which is highly expressed in the central nervous system, which regulates HSP70 machinery and interacts with the proteasome (Parfitt et al., 2009; Anderson et al., 2011).

The fact that our analysis of host-interacting proteins revealed putative targets involved in cell signaling, transcriptional regulation, and vesicle trafficking is of particular interest in the context of *Ehrlichia* pathogenesis. Indeed, recent studies on the cellular biology of *E. chaffeensis* infection demonstrated that some *E. chaffeensis* type I effectors interact with similar eukaryotic proteins (Wakeel et al., 2009; Luo et al., 2011). This reinforces the interest of our approach to identify novel type IV effectors and to facilitate their functional characterization, but could also highlight a possible redundancy of action between type I and type IV effectors of *E. chaffeensis* for better infection.

In summary, our results are in accordance with the current knowledge of *Ehrlichia* molecular pathogenesis (Moumène and Meyer, 2016), and the T4Es we predicted using the S4TE algorithm for *E. chaffeensis* are good candidates for further biological analysis. In addition, the human interactome predicted via HPIDB provides useful information on the possible mode of action of these putative T4Es within the host cell. This study is proof-of-concept that comparative effectomics allows the identification of important host pathways targeted by the bacterial pathogen.

In addition to strain-level variations, allelic diversification in type IV effectors should be further investigated along with variations in regulation or protein expression of these genes. Because type IV effector repertoires are suggested to be major determinants of virulence in *Ehrlichia* (Moumène and Meyer, 2016), it is also important to understand the diversity of type IV effectors present in different species that infect common hosts. Likewise, studying the evolution of type IV effector repertoires among different bacterial species with different host ranges or lifestyles could provide key information to identify the determinants of host specificity.

Based on our results, we hypothesize that the evolution of *E. chaffeensis* intra-species pathogenicity occurs via the acquisition of key regulatory genes. Ultimately, the successive acquisition of type IV effectors could lead to the adaptation of new environmental niches—hosts—resulting in a potential host jump followed by the emergence of new strains in a dynamic environment. However, functional evidence is still lacking for many functions that are hypothetically involved in host specificity.

This study, which focused on type IV effector repertoires in several strains of *E. chaffeensis*, is a step forward in the understanding of *E. chaffeensis* pathobiology. We propose an original approach with rational targets to enable the design of alternative therapies for *ehrlichiae* and other intracellular pathogens.

## Conclusion

Using S4TE software, we predicted *in silico* the putative type IV effectors from available complete genomes among *E. chaffeensis* species. In particular, we searched for proteins with eukaryotic-like domains, signals for addressing organelles, structural features known to be involved in protein-protein interactions or type IV secretion, and homolog to known T4Es in other bacteria. We identified 47 candidate T4Es in *E. chaffeensis* (45 belonging to the core type IV effectome) with several of the above-cited features. Some presented homologies with known type IV effectors in other bacterial systems and others were annotated as hypothetical proteins with no predicted function. We revealed one strain to be a specific candidate effector in the Liberty strain. The majority of predicted T4Es belonged to plastic regions of the genome. Prediction of protein-protein interactions between *E. chaffeensis* T4Es and human proteome revealed host target proteins that could play a critical role in disease development. Experimental characterization of *E. chaffeensis* candidate T4Es and their targets is now required to confirm these predictions. Yet, our study is the first to show the power of comparative effectomics, even in the case of closely related strains at the intra-species level, in deciphering new cellular pathways potentially involved in host-*Anaplasmataceae* interaction.

## Author contributions

CN and DFM conceived the paper, analyzed the results, and wrote the paper.

### Conflict of interest statement

The authors declare that the research was conducted in the absence of any commercial or financial relationships that could be construed as a potential conflict of interest.
