# Corrigendum: Comparative Genomics of the Zoonotic Pathogen *Ehrlichia chaffeensis* Reveals Candidate Type IV Effectors and Putative Host Cell Targets

**DOI:** 10.3389/fcimb.2017.00120

**Published:** 2017-04-04

**Authors:** Christophe Noroy, Damien F. Meyer

**Affiliations:** ^1^CIRAD, UMR ASTREGuadeloupe, France; ^2^INRA, UMR 1309 ASTREMontpellier, France; ^3^Université des AntillesGuadeloupe, France

**Keywords:** type IV effectors, *Ehrlichia chaffeensis*, comparative genomics, host–pathogen interactions, genome plasticity

In the original article, there was a mistake in the legend for **Table 1** as published. The citation of the *Ehrlichia chaffeensis* GSCID genomic project was missing. The correct legend appears below. The authors apologize for this error and state that this does not change the scientific conclusions of the article in any way.

**TABLE 1 | Main biological and genetic characteristics of the eight *Ehrlichia chaffeensis* strains analyzed**. Adapted from Rikihisa et al. (2011).

## Conflict of interest statement

The authors declare that the research was conducted in the absence of any commercial or financial relationships that could be construed as a potential conflict of interest.
