# Supplementary material for: Comparative Genomics of the Zoonotic Pathogen Ehrlichia chaffeensis Reveals Candidate Type IV Effectors and Putative Host Cell Targets
Source: Front Cell Infect Microbiol. 2017 Jan 25;6:204. doi: 10.3389/fcimb.2016.00204 (PMC5263134; doi:10.3389/fcimb.2016.00204)

**Table S1. Homologies of *E. chaffeensis* str. Liberty type IV effectors predicted by S4TE algorithm with known effectors.** This table presents homologies of candidate T4Es identified by S4TE in *E. chaffeensis* str. Liberty with known T4Es.


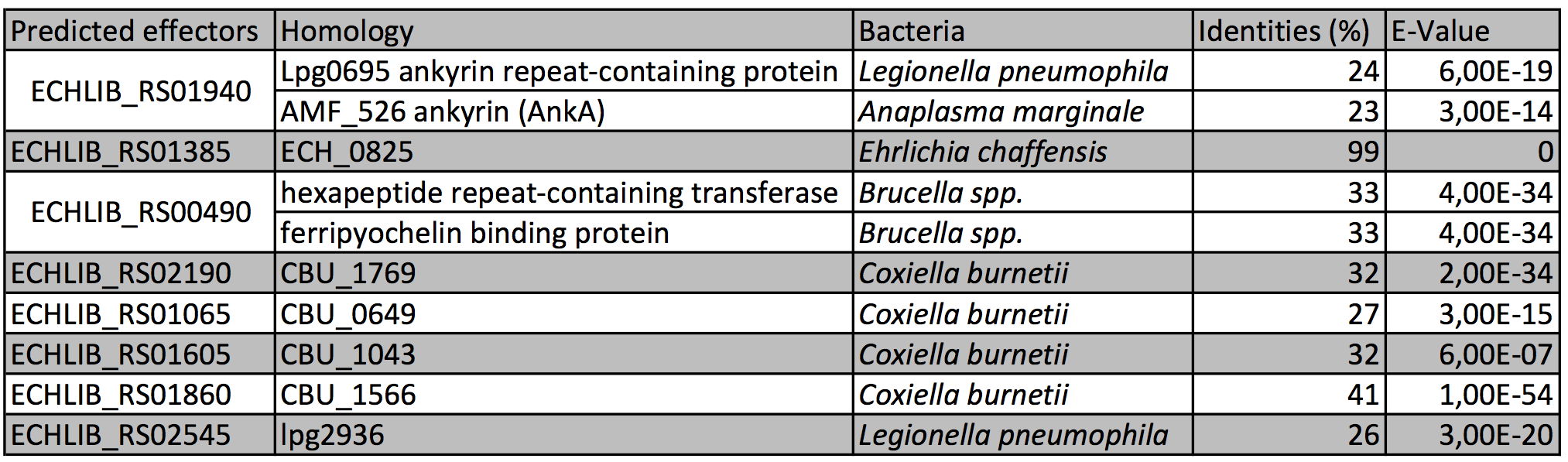

Supplement: Supplementary file 1 [file Table1.DOCX]
