# Supplementary material for: Comparative Genomics of the Zoonotic Pathogen Ehrlichia chaffeensis Reveals Candidate Type IV Effectors and Putative Host Cell Targets
Source: Front Cell Infect Microbiol. 2017 Jan 25;6:204. doi: 10.3389/fcimb.2016.00204 (PMC5263134; doi:10.3389/fcimb.2016.00204)
Supplement: Supplementary file 2 [file Table2.DOCX]

**Table S2. Cellular component, molecular function and biological process of Human proteins interacting with *E. chaffeensis* candidate type IV effectors.** Cellular component, molecular function and biological process were defined by CELLO2GO during the prediction of interactions with *E. chaffeensis* candidate T4Es.

| **Genes** | **Cellular Component** | **Molecular Function** | **Biological Process** |
| --- | --- | --- | --- |
| HMGN2 | Nuclear | DNA binding | chromosome organization |
| CPNS1 | Cytoplasmic | peptidase activity | response to stress |
|  | Nuclear | hydrolase activity | cell differentiation |
|  |  | ion binding | anatomical structure formation involved in morphogenesis |
|  |  | protein binding | anatomical structure development |
|  |  | cytoskeletal protein binding | membrane organization |
|  |  | kinase activity | catabolic process |
|  |  |  | protein maturation |
|  |  |  | transport |
|  |  |  | signal transduction |
|  |  |  | transmembrane transport |
|  |  |  | cell cycle |
|  |  |  | mitosis |
|  |  |  | cell division |
|  |  |  | cellular protein modification process |
| PRR3 | Nuclear | ion binding | biosynthetic process |
|  |  | DNA binding | cellular nitrogen compound metabolic process |
|  |  | RNA binding | protein targeting |
|  |  | protein binding | transport |
|  |  | enzyme binding | nucleocytoplasmic transport |
|  |  | enzyme regulator activity |  |
| APBA1 | Cytoplasmic | lipid binding | protein complex assembly |
|  | Nuclear | ion binding | transport |
|  |  | protein binding | cell adhesion |
|  |  | enzyme regulator activity | cell-cell signaling |
|  |  | signal transducer activity | cellular component assembly |
|  |  | kinase activity | anatomical structure development |
|  |  | structural molecule activity | neurological system process |
|  |  | enzyme binding | macromolecular complex assembly |
|  |  | oxidoreductase activity | embryo development |
|  |  |  | growth |
|  |  |  | vesicle-mediated transport |
|  |  |  | signal transduction |
|  |  |  | cell differentiation |
|  |  |  | locomotion |
|  |  |  | reproduction |
|  |  |  | developmental maturation |
|  |  |  | cell death |
|  |  |  | membrane organization |
|  |  |  | biosynthetic process |
|  |  |  | cellular nitrogen compound metabolic process |
| ZN720 | Cytoplasmic | DNA binding | biosynthetic process |
|  |  | ion binding | cellular nitrogen compound metabolic process |
|  |  | nucleic acid binding transcription factor activity |  |
| ZN227 | Nuclear | DNA binding | biosynthetic process |
|  |  | ion binding | cellular nitrogen compound metabolic process |
|  |  | nucleic acid binding transcription factor activity |  |
| DPYD | Cytoplasmic | protein binding | cellular amino acid metabolic process |
|  |  | oxidoreductase activity | catabolic process |
|  |  | ion binding | biosynthetic process |
|  |  |  | cellular nitrogen compound metabolic process |
|  |  |  | nucleobase-containing compound catabolic process |
|  |  |  | small molecule metabolic process |
|  |  |  | growth |
| HG2A | Extracellular | signal transducer activity | cellular protein modification process |
|  | Nuclear | protein binding | transport |
|  |  | enzyme binding | signal transduction |
|  |  | ion binding | biosynthetic process |
|  |  | enzyme regulator activity | cellular component assembly |
|  |  |  | cell differentiation |
|  |  |  | small molecule metabolic process |
|  |  |  | anatomical structure development |
|  |  |  | cell adhesion |
|  |  |  | extracellular matrix organization |
|  |  |  | sulfur compound metabolic process |
|  |  |  | locomotion |
|  |  |  | cell motility |
|  |  |  | homeostatic process |
|  |  |  | cellular amino acid metabolic process |
| ARI4B | Nuclear | DNA binding | biosynthetic process |
|  |  | nucleic acid binding transcription factor activity | cellular nitrogen compound metabolic process |
|  |  | protein binding transcription factor activity | cellular protein modification process |
|  |  | ion binding | chromosome organization |
|  |  | oxidoreductase activity | reproduction |
|  |  |  | cell differentiation |
|  |  |  | locomotion |
|  |  |  | anatomical structure development |
|  |  |  | signal transduction |
| COA6 | Extracellular | oxidoreductase activity | protein complex assembly |
|  | Nuclear | transmembrane transporter activity | mitochondrion organization |
|  |  |  | cellular component assembly |
|  |  |  | macromolecular complex assembly |
|  |  |  | generation of precursor metabolites and energy |
| CD4 | Extracellular | signal transducer activity | reproduction |
|  | Nuclear | structural molecule activity | immune system process |
|  |  | protein binding | cellular protein modification process |
|  |  | ion binding | response to stress |
|  |  | oxidoreductase activity | cell adhesion |
|  |  |  | signal transduction |
|  |  |  | biosynthetic process |
|  |  |  | cell differentiation |
|  |  |  | locomotion |
|  |  |  | symbiosis, encompassing mutualism through parasitism |
|  |  |  | anatomical structure development |
|  |  |  | catabolic process |
|  |  |  | extracellular matrix organization |
| FKBP8 | Cytoplasmic | isomerase activity | reproduction |
|  | Nuclear | protein binding | protein folding |
|  |  | ion binding | signal transduction |
|  |  | hydrolase activity | cell differentiation |
|  |  |  | anatomical structure development |
|  |  |  | cellular protein modification process |
|  |  |  | response to stress |
|  |  |  | transport |
|  |  |  | small molecule metabolic process |
|  |  |  | homeostatic process |
|  |  |  | chromosome organization |
| NFKB1 | Cytoplasmic | nucleic acid binding transcription factor activity | immune system process |
|  |  | DNA binding | response to stress |
|  |  | protein binding | signal transduction |
|  |  | ion binding | cell death |
|  |  | enzyme binding | biosynthetic process |
|  |  | ligase activity | cellular nitrogen compound metabolic process |
|  |  | kinase activity | anatomical structure development |
|  |  | transferase activity, transferring acyl groups | cellular protein modification process |
|  |  | transmembrane transporter activity | cell differentiation |
|  |  | lipid binding | reproduction |
|  |  | enzyme regulator activity | embryo development |
|  |  | hydrolase activity | transport |
|  |  | RNA binding | vesicle-mediated transport |
|  |  |  | cell cycle |
|  |  |  | neurological system process |
| CALR | ER | protein binding | protein folding |
|  |  | ion binding | reproduction |
|  |  | unfolded protein binding | immune system process |
|  |  | RNA binding | protein complex assembly |
|  |  | mRNA binding | transport |
|  |  |  | response to stress |
|  |  |  | aging |
|  |  |  | cellular component assembly |
|  |  |  | cell differentiation |
|  |  |  | homeostatic process |
|  |  |  | anatomical structure development |
|  |  |  | macromolecular complex assembly |
|  |  |  | neurological system process |
|  |  |  | cell death |
|  |  |  | vesicle-mediated transport |
|  |  |  | cell-cell signaling |
|  |  |  | catabolic process |
| KAP2 | Cytoplasmic | protein binding | transport |
|  | Nuclear | enzyme binding | response to stress |
|  |  | enzyme regulator activity | signal transduction |
|  |  | ion binding | small molecule metabolic process |
|  |  | kinase activity | transmembrane transport |
|  |  | hydrolase activity | lipid metabolic process |
|  |  | transmembrane transporter activity | neurological system process |
|  |  |  | cellular protein modification process |
|  |  |  | anatomical structure development |
|  |  |  | cell differentiation |
|  |  |  | locomotion |
|  |  |  | embryo development |
|  |  |  | anatomical structure formation involved in morphogenesis |
|  |  |  | reproduction |
|  |  |  | cell motility |
| CO4A1 | Extracellular | structural molecule activity | cell morphogenesis |
|  | Nuclear | protein binding | cell differentiation |
|  |  | enzyme regulator activity | extracellular matrix organization |
|  |  | ion binding | locomotion |
|  |  |  | anatomical structure formation involved in morphogenesis |
|  |  |  | anatomical structure development |
|  |  |  | cell adhesion |
|  |  |  | signal transduction |
|  |  |  | neurological system process |
|  |  |  | transport |
|  |  |  | embryo development |
|  |  |  | immune system process |
|  |  |  | response to stress |
|  |  |  | homeostatic process |
|  |  |  | protein complex assembly |
|  |  |  | cellular component assembly |
|  |  |  | macromolecular complex assembly |
|  |  |  | cell junction organization |
| GSTP1 | Extracellular | protein binding | cellular amino acid metabolic process |
|  | Cytoplasmic | transferase activity, transferring alkyl or aryl (other than methyl) groups | sulfur compound metabolic process |
|  |  | ion binding | response to stress |
|  |  | structural molecule activity | cellular nitrogen compound metabolic process |
|  |  | isomerase activity | small molecule metabolic process |
|  |  |  | aging |
|  |  |  | catabolic process |
|  |  |  | lipid metabolic process |
|  |  |  | biosynthetic process |
| IRF9 | Nuclear | nucleic acid binding transcription factor activity | immune system process |
|  |  | DNA binding | response to stress |
|  |  | methyltransferase activity | signal transduction |
|  |  | protein binding | biosynthetic process |
|  |  | protein binding transcription factor activity | cellular nitrogen compound metabolic process |
|  |  |  | transport |
|  |  |  | vesicle-mediated transport |
|  |  |  | cell differentiation |
|  |  |  | anatomical structure development |
|  |  |  | cellular protein modification process |
|  |  |  | cell cycle |
|  |  |  | cell proliferation |
|  |  |  | reproduction |
| TIE2 | Cytoplasmic | signal transducer activity | cell morphogenesis |
|  | Nuclear | kinase activity | immune system process |
|  |  | ion binding | cellular protein modification process |
|  |  | protein binding | response to stress |
|  |  | lipid binding | cell adhesion |
|  |  |  | signal transduction |
|  |  |  | cell-cell signaling |
|  |  |  | cell proliferation |
|  |  |  | cell differentiation |
|  |  |  | locomotion |
|  |  |  | anatomical structure formation involved in morphogenesis |
|  |  |  | anatomical structure development |
|  |  |  | cell motility |
|  |  |  | embryo development |
|  |  |  | cell death |
|  |  |  | homeostatic process |
|  |  |  | neurological system process |
|  |  |  | reproduction |
|  |  |  | transport |
|  |  |  | vesicle-mediated transport |
| Z518A | Nuclear | DNA binding | biosynthetic process |
|  |  | ion binding | cellular nitrogen compound metabolic process |
|  |  | nucleic acid binding transcription factor activity | cell differentiation |
|  |  | protein binding | anatomical structure development |
|  |  |  | reproduction |
| TSNAX | Nuclear | DNA binding | reproduction |
|  |  | ion binding | cell differentiation |
|  |  | protein transporter activity | cellular nitrogen compound metabolic process |
|  |  | protein binding | DNA metabolic process |
|  |  | RNA binding |  |
|  |  | nuclease activity |  |
|  |  | hydrolase activity |  |
|  |  | mRNA binding |  |
| N42L2 | Nuclear | nuclease activity | embryo development |
|  |  | kinase activity | anatomical structure development |
|  |  | hydrolase activity | cell morphogenesis |
|  |  | ion binding | cytoskeleton organization |
|  |  | RNA binding | catabolic process |
|  |  |  | cell differentiation |
|  |  |  | cellular nitrogen compound metabolic process |
|  |  |  | nucleobase-containing compound catabolic process |
|  |  |  | small molecule metabolic process |
| HERP2 | Plasmamembrane | N/A | reproduction |
|  | Nuclear |  | response to stress |
|  |  |  | signal transduction |
|  |  |  | cell death |
|  |  |  | catabolic process |
|  |  |  | homeostatic process |
| SDCB1 | Mitochondrial | protein binding | transport |
|  |  | cytoskeletal protein binding | cytoskeleton organization |
|  |  | protein binding, bridging | signal transduction |
|  |  | enzyme regulator activity | cell-cell signaling |
|  |  | lipid binding | cell differentiation |
|  |  | ion binding | locomotion |
|  |  | enzyme binding | anatomical structure development |
|  |  | kinase activity | neurological system process |
|  |  |  | embryo development |
|  |  |  | cell adhesion |
|  |  |  | vesicle-mediated transport |
|  |  |  | growth |
|  |  |  | cell proliferation |
|  |  |  | developmental maturation |
|  |  |  | membrane organization |
| DDX5 | Nuclear | RNA binding | mRNA processing |
|  |  | helicase activity | biosynthetic process |
|  |  | hydrolase activity | cellular nitrogen compound metabolic process |
|  |  | ATPase activity | catabolic process |
|  |  | ion binding | nucleobase-containing compound catabolic process |
|  |  | translation factor activity, nucleic acid binding | ribosome biogenesis |
|  |  |  | translation |
|  |  |  | reproduction |
| CO1A2 | Extracellular | structural molecule activity | cell morphogenesis |
|  | Nuclear | protein binding | immune system process |
|  |  | protein binding, bridging | protein complex assembly |
|  |  | ion binding | response to stress |
|  |  | signal transducer activity | signal transduction |
|  |  |  | cellular component assembly |
|  |  |  | cell differentiation |
|  |  |  | extracellular matrix organization |
|  |  |  | locomotion |
|  |  |  | anatomical structure development |
|  |  |  | macromolecular complex assembly |
|  |  |  | cell adhesion |
|  |  |  | embryo development |
|  |  |  | homeostatic process |
|  |  |  | anatomical structure formation involved in morphogenesis |
|  |  |  | neurological system process |
|  |  |  | transport |
|  |  |  | biosynthetic process |
| WBS22 | Cytoplasmic | methyltransferase activity | transport |
|  | Nuclear |  | nucleocytoplasmic transport |
|  |  |  | cell cycle |
|  |  |  | cellular nitrogen compound metabolic process |
|  |  |  | ribosome biogenesis |
|  |  |  | cell division |
| DPM1 | Mitochondrial | transferase activity, transferring glycosyl groups | carbohydrate metabolic process |
|  | Chloroplast |  | cellular protein modification process |
|  |  |  | lipid metabolic process |
|  |  |  | biosynthetic process |
|  |  |  | cellular nitrogen compound metabolic process |
|  |  |  | small molecule metabolic process |
| IDHC | Cytoplasmic | oxidoreductase activity | carbohydrate metabolic process |
|  |  | ion binding | generation of precursor metabolites and energy |
|  |  | protein binding | cellular amino acid metabolic process |
|  |  |  | sulfur compound metabolic process |
|  |  |  | response to stress |
|  |  |  | cellular nitrogen compound metabolic process |
|  |  |  | small molecule metabolic process |
|  |  |  | cofactor metabolic process |
|  |  |  | biosynthetic process |
| IGKC | Extracellular | N/A | immune system process |
|  |  |  | response to stress |
| ZN627 | Nuclear | DNA binding | biosynthetic process |
|  |  | ion binding | cellular nitrogen compound metabolic process |
|  |  | nucleic acid binding transcription factor activity |  |
| CNTRL | Cytoplasmic | protein binding | cell cycle |
|  | Cytoskeletal | kinase activity | cell division |
|  | Nuclear | ion binding | mRNA processing |
|  |  | RNA binding | cellular nitrogen compound metabolic process |
|  |  |  | cell morphogenesis |
|  |  |  | anatomical structure development |
|  |  |  | reproduction |
|  |  |  | mitosis |
| SYVN1 | Plasmamembrane | ligase activity | cellular protein modification process |
|  |  | ion binding | response to stress |
|  |  | protein binding | signal transduction |
|  |  | DNA binding | catabolic process |
|  |  |  | biosynthetic process |
|  |  |  | embryo development |
|  |  |  | anatomical structure development |
|  |  |  | neurological system process |
|  |  |  | immune system process |
|  |  |  | cell death |
|  |  |  | cell differentiation |
| PUF60 | Cytoplasmic | DNA binding | mRNA processing |
|  |  | RNA binding | biosynthetic process |
|  |  | mRNA binding | cellular nitrogen compound metabolic process |
|  |  | translation factor activity, nucleic acid binding | reproduction |
|  |  | isomerase activity | cellular component assembly |
|  |  | ion binding | cell differentiation |
|  |  |  | anatomical structure development |
|  |  |  | macromolecular complex assembly |
|  |  |  | transport |
|  |  |  | translation |
|  |  |  | response to stress |
|  |  |  | catabolic process |
|  |  |  | nucleobase-containing compound catabolic process |
|  |  |  | ribosome biogenesis |
|  |  |  | protein folding |
|  |  |  | cellular protein modification process |
| FYN | Cytoplasmic | kinase activity | reproduction |
|  |  | ion binding | cell morphogenesis |
|  |  | protein binding | immune system process |
|  |  | lipid binding | cellular protein modification process |
|  |  | signal transducer activity | transport |
|  |  |  | response to stress |
|  |  |  | signal transduction |
|  |  |  | cell proliferation |
|  |  |  | cell differentiation |
|  |  |  | locomotion |
|  |  |  | anatomical structure development |
|  |  |  | cell motility |
|  |  |  | neurological system process |
|  |  |  | homeostatic process |
|  |  |  | embryo development |
|  |  |  | cell adhesion |
|  |  |  | cell death |
|  |  |  | vesicle-mediated transport |
|  |  |  | cell-cell signaling |
|  |  |  | cytoskeleton organization |
|  |  |  | anatomical structure formation involved in morphogenesis |
| IF4E2 | Nuclear | RNA binding | signal transduction |
|  |  | translation factor activity, nucleic acid binding | embryo development |
|  |  | protein binding | anatomical structure development |
|  |  | lipid binding | translation |
|  |  |  | biosynthetic process |
|  |  |  | cell cycle |
|  |  |  | reproduction |
|  |  |  | cell differentiation |
|  |  |  | cellular nitrogen compound metabolic process |
|  |  |  | catabolic process |
|  |  |  | nucleobase-containing compound catabolic process |
|  |  |  | response to stress |
| PGS1 | Extracellular | structural molecule activity | cellular protein modification process |
|  | Cytoplasmic | ion binding | sulfur compound metabolic process |
|  |  | protein binding | small molecule metabolic process |
|  |  | signal transducer activity | anatomical structure development |
|  |  | enzyme regulator activity | response to stress |
|  |  | RNA binding | cell adhesion |
|  |  | kinase activity | neurological system process |
|  |  | enzyme binding | reproduction |
|  |  | transmembrane transporter activity | signal transduction |
|  |  |  | cell differentiation |
|  |  |  | cell morphogenesis |
|  |  |  | immune system process |
| PTK7 | Cytoplasmic | signal transducer activity | response to stress |
|  | Nuclear | kinase activity | cell adhesion |
|  |  | ion binding | signal transduction |
|  |  | protein binding | embryo development |
|  |  | lipid binding | cell differentiation |
|  |  |  | locomotion |
|  |  |  | anatomical structure formation involved in morphogenesis |
|  |  |  | anatomical structure development |
|  |  |  | cell motility |
|  |  |  | cellular protein modification process |
|  |  |  | cell death |
|  |  |  | cell morphogenesis |
|  |  |  | cell-cell signaling |
|  |  |  | cell proliferation |
|  |  |  | neurological system process |
|  |  |  | homeostatic process |
|  |  |  | reproduction |
|  |  |  | immune system process |
|  |  |  | transport |
| RRAGB | Nuclear | ion binding | signal transduction |
|  |  | protein binding | reproduction |
|  |  | GTPase activity | cell death |
|  |  | hydrolase activity | symbiosis, encompassing mutualism through parasitism |
|  |  |  | transport |
|  |  |  | biosynthetic process |
|  |  |  | cellular nitrogen compound metabolic process |
|  |  |  | catabolic process |
|  |  |  | membrane organization |
|  |  |  | growth |
| LRC45 | Extracellular | ion binding | immune system process |
|  |  | protein binding | signal transduction |
|  |  | enzyme binding | protein complex assembly |
|  |  | enzyme regulator activity | cellular protein modification process |
|  |  | DNA binding | response to stress |
|  |  |  | cellular component assembly |
|  |  |  | macromolecular complex assembly |
|  |  |  | transport |
|  |  |  | cell cycle |
|  |  |  | reproduction |
|  |  |  | cell differentiation |
|  |  |  | anatomical structure development |
| RN125 | Extracellular | ligase activity | immune system process |
|  | Nuclear | ion binding | response to stress |
|  |  | protein binding | cellular protein modification process |
|  |  | signal transducer activity | reproduction |
|  |  | DNA binding | cell differentiation |
|  |  | histone binding | signal transduction |
|  |  | helicase activity | catabolic process |
|  |  | hydrolase activity | anatomical structure development |
|  |  | ATPase activity | DNA metabolic process |
|  |  | peptidase activity | cell cycle |
|  |  |  | biosynthetic process |
|  |  |  | cellular nitrogen compound metabolic process |
|  |  |  | chromosome organization |
|  |  |  | cell death |
| TINAL | Extracellular | peptidase activity | immune system process |
|  |  | hydrolase activity | response to stress |
|  |  | protein binding | catabolic process |
|  |  |  | anatomical structure development |
|  |  |  | aging |
|  |  |  | homeostatic process |
|  |  |  | cell differentiation |
|  |  |  | pathogenesis |
|  |  |  | cell death |
| ATM | Nuclear | DNA binding | reproduction |
|  |  | protein binding | DNA metabolic process |
|  |  | kinase activity | cellular protein modification process |
|  |  | ion binding | response to stress |
|  |  | protein binding transcription factor activity | cell cycle |
|  |  |  | signal transduction |
|  |  |  | aging |
|  |  |  | catabolic process |
|  |  |  | embryo development |
|  |  |  | cell differentiation |
|  |  |  | cellular nitrogen compound metabolic process |
|  |  |  | nucleobase-containing compound catabolic process |
|  |  |  | homeostatic process |
|  |  |  | anatomical structure development |
|  |  |  | chromosome organization |
|  |  |  | biosynthetic process |
|  |  |  | transport |
|  |  |  | locomotion |
|  |  |  | vesicle-mediated transport |
|  |  |  | lipid metabolic process |
|  |  |  | cell division |
| TRPC1 | Plasmamembrane | transmembrane transporter activity | cell morphogenesis |
|  |  | ion binding | transport |
|  |  | protein binding | cell differentiation |
|  |  | DNA binding | locomotion |
|  |  | lipid binding | homeostatic process |
|  |  | hydrolase activity | anatomical structure development |
|  |  |  | transmembrane transport |
|  |  |  | cell-cell signaling |
|  |  |  | neurological system process |
|  |  |  | response to stress |
|  |  |  | signal transduction |
| MK14 | Cytoplasmic | signal transducer activity | immune system process |
|  | Mitochondrial | protein binding | cellular protein modification process |
|  |  | kinase activity | response to stress |
|  |  | ion binding | cell cycle |
|  |  |  | signal transduction |
|  |  |  | cell death |
|  |  |  | biosynthetic process |
|  |  |  | cell differentiation |
|  |  |  | cellular nitrogen compound metabolic process |
|  |  |  | anatomical structure development |
|  |  |  | cell division |
|  |  |  | reproduction |
|  |  |  | mitosis |
| Q53TK7 | Cytoplasmic | hydrolase activity | N/A |
|  | Nuclear |  |  |
| BCLF1 | Nuclear | DNA binding | response to stress |
|  |  | protein binding transcription factor activity | biosynthetic process |
|  |  | protein binding | cellular nitrogen compound metabolic process |
|  |  | transcription factor binding | signal transduction |
|  |  | ion binding | catabolic process |
|  |  |  | nucleobase-containing compound catabolic process |
|  |  |  | mRNA processing |
| SACS | Cytoplasmic | protein binding | protein folding |
|  | Nuclear | ion binding | cell death |
| AMRA1 | Nuclear | protein binding | catabolic process |
|  |  | histone binding | cell differentiation |
|  |  | transferase activity, transferring acyl groups | anatomical structure development |
|  |  | cytoskeletal protein binding | cellular protein modification process |
|  |  | structural molecule activity | chromosome organization |
|  |  | protein binding transcription factor activity | biosynthetic process |
|  |  | nucleic acid binding transcription factor activity | cellular nitrogen compound metabolic process |
|  |  | signal transducer activity | cell cycle |
|  |  | RNA binding | mitosis |
|  |  | DNA binding | cell division |
|  |  |  | response to stress |
|  |  |  | reproduction |
|  |  |  | transport |
|  |  |  | vesicle-mediated transport |
|  |  |  | signal transduction |
|  |  |  | ribosome biogenesis |
| AL1A1 | Cytoplasmic | lipid binding | lipid metabolic process |
|  |  | oxidoreductase activity | small molecule metabolic process |
|  |  | protein binding | response to stress |
|  |  | ion binding | biosynthetic process |
|  |  | methyltransferase activity | anatomical structure development |
|  |  |  | catabolic process |
|  |  |  | cellular nitrogen compound metabolic process |
|  |  |  | cofactor metabolic process |
|  |  |  | cellular amino acid metabolic process |
| TRI14 | Mitochondrial | ion binding | cellular protein modification process |
|  | Nuclear | protein binding | cell death |
|  |  | ligase activity | immune system process |
|  |  | signal transducer activity | cytoskeleton organization |
|  |  | DNA binding | biosynthetic process |
|  |  |  | cellular nitrogen compound metabolic process |
|  |  |  | response to stress |
|  |  |  | lipid metabolic process |
|  |  |  | transport |
|  |  |  | cell cycle |
|  |  |  | cell differentiation |
|  |  |  | anatomical structure development |
|  |  |  | chromosome organization |
| CA174 | Nuclear | N/A | N/A |
| LENG8 | Nuclear | DNA binding | transport |
|  |  |  | nucleocytoplasmic transport |
|  |  |  | biosynthetic process |
|  |  |  | cellular nitrogen compound metabolic process |
|  |  |  | mRNA processing |
|  |  |  | DNA metabolic process |
|  |  |  | protein targeting |
| INF2 | Nuclear | protein binding | cytoskeleton organization |
|  |  | cytoskeletal protein binding | cellular component assembly |
|  |  | enzyme binding | cell differentiation |
|  |  | hydrolase activity | anatomical structure development |
|  |  | phosphatase activity | reproduction |
|  |  | DNA binding | cell division |
|  |  |  | cell cycle |
|  |  |  | signal transduction |
|  |  |  | locomotion |
|  |  |  | cell motility |
